# SAID: Segment All Industrial Defects with Scene Prompts

**DOI:** 10.3390/s25164929

**Published:** 2025-08-09

**Authors:** Yican Huang, Junwei Zhu, Xiaopin Zhong, Yuanlong Deng

**Affiliations:** 1 College of Mechatronics and Control Engineering, Shenzhen University, Nanhai Ave., Shenzhen 518060, China; huangyican2022@email.szu.edu.cn; 2 School of Sino-Germany Intelligent Production, Shenzhen City Polytechnic, Shenzhen 518116, China; zhujunwei@szcp.edu.cn (J.Z.); dengyl@szu.edu.cn (Y.D.); 3 College of Urban Transportation and Logistics, Shenzhen Technology University, Shenzhen 518118, China

**Keywords:** industrial defect segmentation, cross-scene adaptation, prompt-based foundation model

## Abstract

In the field of industrial inspection, image segmentation is a common method for surface inspection, capable of locating and segmenting the appearance defect areas of products. Most existing methods are trained specifically for particular products. The recent SAM (Segment Anything Model) serves as an image segmentation foundation model, capable of achieving zero-shot segmentation through diverse prompts. Nevertheless, SAM’s performance in special downstream tasks is not satisfactory. Additionally, SAM requires prior manual interactions to complete segmentation and post-processing of the segmentation results. This paper proposes SAID (Segment All Industrial Defects) to deal with these issues. The SAID model encodes single-annotated prompt–image pairs into scene embedding via Scene Encoder, achieving automatic segmentation and eliminating the reliance on manual intervention. Meanwhile, SAID’s Feature Alignment and Fusion Module effectively addresses the alignment issue between scene embedding and image embedding. Experimental results demonstrate that SAID outperforms SAM in segmentation capabilities across various industrial scenes. Under the one-shot target scene segmentation task, SAID also improves the mIoU metrics by 5.79 and 0.87 compared to the MSNet and SegGPT, respectively.

## 1. Introduction

Industrial surface defect detection is a critical quality control technology aimed at identifying appearance defects on surfaces, such as scratches, dirt, and damage. Image segmentation transforms the surface defect detection task into a semantic segmentation problem between defective and normal regions, enabling fine segmentation of defect areas and obtaining their location and geometric attributes. Currently, industrial image segmentation methods based on deep learning can be divided into two categories: one is professional models, which usually use supervised methods for training from scratch; the other is to use the foundation model and employ prompt learning mechanism for segmentation.

For the first, although professional segmentation methods like Mask-RCNN [1], DeepLabV3 [2], and SegFormer [3] have achieved significant success in the field of image segmentation, the segmentation task in industrial surface defect detection still faces several challenges: (1) Lack of defect samples [4]. Normal samples of industrial products are easy to obtain, but defect samples are relatively scarce. (2) Numerous scene types and complex defect patterns [5], such as surface defects of industrial products exhibiting various shapes, colors, textures, etc. (3) Ambiguous defect evaluation criteria. Industrial products are prone to undefined defect types, posing challenges to traditional supervised learning-based segmentation methods.

For the second, with the development of computing power and large-scale datasets, foundation models [6] continue to emerge. Recently, Meta AI’s foundational visual segmentation model SAM [7] has garnered attention for its ability to generate precise object masks in an interactive manner. SAM is trained on the large-scale dataset SA-1B, possessing powerful zero-shot generalization capability. However, due to significant differences between natural images and downstream task images from other fields (such as industrial images, medical images [8], and remote sensing images [9]), directly using SAM for segmentation in downstream fine-grained fields does not yield satisfactory results. Notably, SAM requires manual interaction prompts, which poses real-time issues in practical applications.

**Figure 1:**
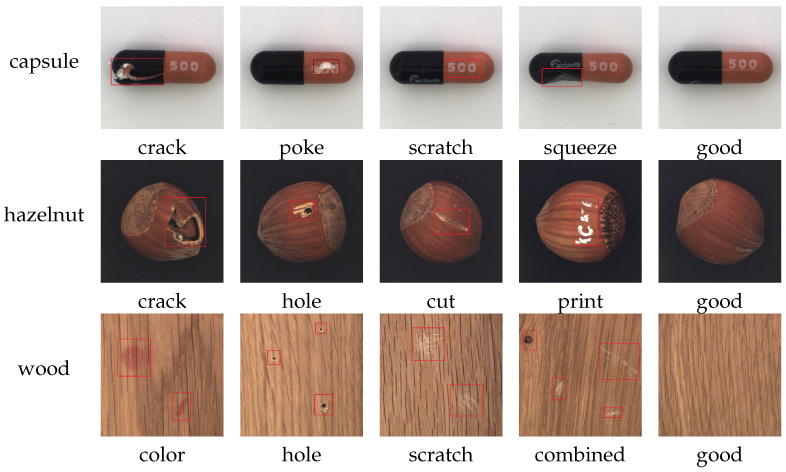
Multiple products in the industrial field have various defects.

**Figure 2:**
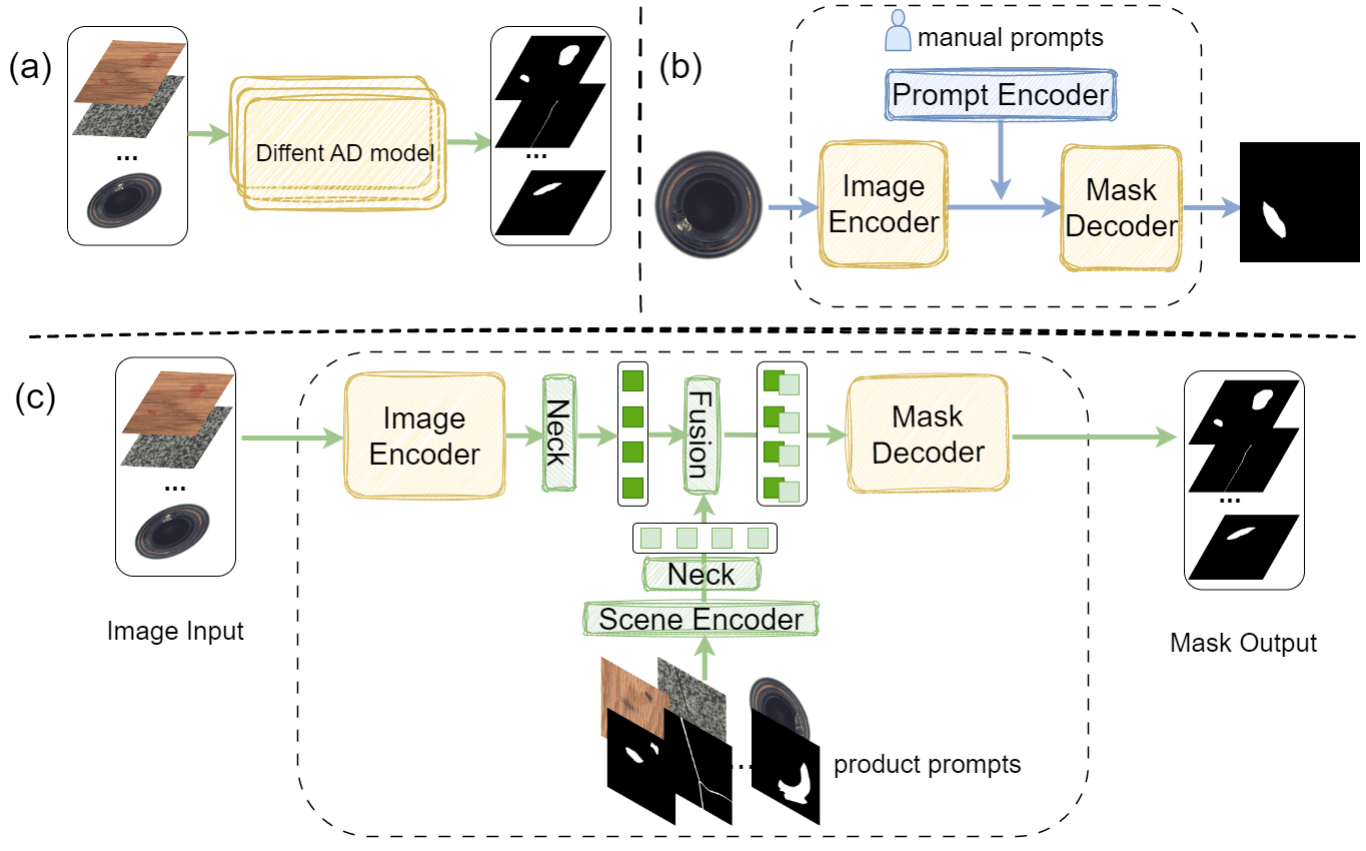
Illustration of the differences between traditional methods (**a**), SAM-based methods (**b**), and the proposed SAID framework (**c**).

In summary, traditional deep learning methods have poor generalization across different scenes, while SAM lacks capability in downstream industrial fields, and its prompt mechanism is not suitable for industrial detection. To develop SAM’s powerful automatic segmentation capability with cross-scene transferability on industrial images, we design the network SAID for industrial cross-scene single-annotation prompt segmentation. (1) SAID utilizes scene embedding without real-time human interaction. As shown in Figure 1, the input for the scene prompt is a set of product single-annotated prompt–image pairs, which the model encodes into the scene prompt to assist with segmentation. SAID possesses generalization capability for industrial scene images, requiring only the product image and the corresponding annotation mask as the product prompt–image pair to complete the segmentation of abnormal regions. (2) We note that the introduction of the Scene Encoder module inevitably leads to feature alignment issues between image embedding and scene embedding. We design a Lightweight Feature Alignment and Fusion Module, including the Neck module and Lightweight Fusion module, to effectively address this issue. As illustrated in Figure 2, traditional industrial defect detection models typically require training dedicated models for each specific scene, which significantly limits their generalization capability. In contrast, SAID exhibits robust cross-scene generalization by leveraging product prompt pairs—each consisting of a product image and its corresponding annotation—from a given scene to form a transferable scene prompt. While SAM relies on manual interaction to generate segmentation prompts, SAID eliminates this need by automatically encoding the prompt pairs through the Scene Encoder, enabling effective segmentation in unseen scenes without additional human input. Our code will be publicly available at the following URL: https://github.com/KLIVIS/SAID-IVD (accessed on 24 July 2025).

The main contributions of our work are as follows:

1. We propose SAID for the defect segmentation task of industrial images. SAID eliminates the reliance on human priors and does not require complex post-processing, achieving automatic defect segmentation detection.
2. We design the Scene Encoder to encode the product with a set of user-input product annotation images into scene embedding, enhancing the model’s segmentation capabilities. To address the misalignment of features between the Scene Encoder and Image Encoder outputs, one Lightweight Feature Alignment and Fusion Module is designed.
3. Experiments on multiple industrial scene datasets show that our SAID model exhibits excellent capabilities under both one-shot and supervised settings.

## 2. Related Work

### 2.1. Surface Defect Detection

Surface defect detection is a significant technology in the field of computer vision. Traditional vision-based defect detection methods [10,11,12] primarily relied on handcrafted features such as visual textures and colors to detect defects, which perform poorly for complex backgrounds and lighting variations. In recent years, deep learning-based segmentation methods have become the preferred task for surface defect detection. These methods can be categorized into two types: supervised learning-based methods and unsupervised learning-based methods. Supervised learning-based methods require a large amount of annotated data to train models, such as commonly used models like FCN [13] and Mask RCNN [1]. These methods perform well when there is sufficient annotated data, but obtaining large amounts of annotated data is often costly and time-consuming. However, unsupervised learning-based methods do not need annotated data and are mainly divided into reconstruction-based methods and embedding-based methods. Reconstruction-based methods [14,15] use reconstruction networks to reconstruct normal images, and abnormal images are easily detected as they cannot be well reconstructed [16,17]. Embedding-based methods [18,19,20] typically use deep neural networks to pre-train feature extractors on large datasets such as ImageNet [21], extracting meaningful vectors that describe the entire image, with anomaly scores usually represented by the distance between the embedding vector of the test image and the reference vector representing the normality of the training dataset. These unsupervised methods provide an effective solution in the absence of annotated data.

### 2.2. Few-Shot Image Segmentation Methods

In order to address the issue of limited annotated samples for segmentation, some researchers have proposed various few-shot image segmentation methods. These methods can be primarily categorized into meta-learning [22,23], Siamese Network-based [24,25], and prompt learning [26] approaches. Compared to unsupervised methods, few-shot learning provides the model with a clear learning objective to achieve better model performance. Meta-learning enables the model to quickly learn and adapt to new tasks from a small number of samples. By pre-learning some general knowledge or parameters, the model can better adapt to segmentation tasks of different categories. Siamese Network-based methods compare the target image with a few samples to learn a general feature representation. The Siamese Network can quickly adapt on a few samples, thereby achieving accurate segmentation. The core of prompt learning methods lies in designing clever templates that make the training approach for downstream fine-tuning tasks more similar to the pre-training tasks [27]. This design helps to reduce the semantic gap between pre-training and fine-tuning, making the training process more effective and efficient. However, prompt learning requires a foundation model with sufficiently strong emergent capabilities.

### 2.3. Fundamental Visual Segmentation Model

SAM [7] is a foundation model designed by Meta AI for image segmentation tasks. Its architecture is based on Transformer [28], including an Image Encoder, a Prompt Encoder, and a Mask Decoder. The Image Encoder, using ViT (Vision Transformer) [29] as its backbone, is pre-trained with MAE (Masked Autoencoder) [30] to encode the input image into image embedding. The Prompt Encoder consists of dense and sparse branches. SAM is trained on a large-scale annotated dataset, SA-1B, and demonstrates robust zero-shot generalization capability. However, despite its excellent performance on natural images, SAM’s effectiveness in downstream specialized tasks such as agriculture, remote sensing, and medical imaging is poor [8,31,32]. To enhance the performance of SAM in downstream specialized tasks, researchers have proposed various methods. Among these, PEFT (Prompt-based Evolutionary Fine-tuning) [33] is a common and effective technique. PEFT allows for fine-tuning SAM’s performance in specific domains, as demonstrated by studies like SAM-Adapter [34] and Medical Sam Adapter [35]. Additionally, SonarSAM [36] introduces SAM into the field of sonar images and enhances its performance through LoRA [37] technology. Beyond fine-tuning techniques, some works have attempted to modify the prompt network to improve SAM. RSPrompter [9] integrates human prompts into the network itself, achieving superior segmentation performance on remote sensing datasets. SEEM [38], on the other hand, is a model employing a universal encoder–decoder architecture, providing new insights into addressing the performance issues of foundational segmentation models across various downstream tasks through complex query and prompt interactions.

## 3. Methods

Traditional deep learning-based defect detection methods [14,39,40] exhibit poor generalization across different scenes. They often require expert models to be trained for each distinct scene. SAM, while effective in some contexts, suffers in specialized industrial domains due to its need for manual interaction and tendency to generate excessive redundant masks. Building upon SAM, we introduces a Scene Encoder and a Lightweight Feature Alignment and Fusion Module to construct a model named SAID, which can perform automatic segmentation on industrial data using single-annotation information.

### 3.1. Overview of the SAID Architecture

**Figure 3:**
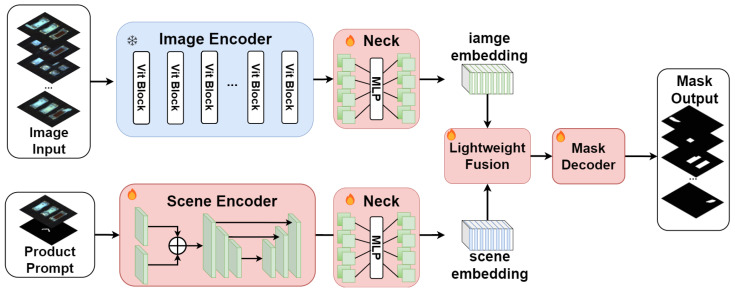
The SAID model architecture consists of an Image Encoder in the upper part, which is frozen and does not participate in training. The Scene Encoder in the lower part requires training.

The network SAID consists of four main components: Image Encoder, Scene Encoder, Feature Alignment and Fusion Module, and Mask Decoder. Figure 3 illustrates the model architecture of SAID. The Image Encoder, adopted from SAM, is frozen during training and not subject to parameter updates. The Scene Encoder is designed to encode a pair of annotated product images into scene embedding, aiding SAID in achieving automatic segmentation without additional human intervention. The Feature Alignment and Fusion Module efficiently integrates feature maps from both the Image Encoder and the Scene Encoder before feeding them into the decoder for mask prediction. The Mask Decoder in SAID is identical to that in SAM and is fine-tuned during training.

The workflow of the model includes the following steps:

(1). The image to be detected $I_{input}\;\in \;R^{3\times H\times W}$ is encoded by the Image Encoder to produce the image embedding $E_{image}\;\in \;R^{256\times 64\times 64}$.
(2). Scene Encoder encodes a pair of product images $I\_sample\;\in \;R^{3\times 1024\times 1024}$ and their corresponding mask images $M\_sample\;\in \;R^{3\times 1024\times 1024}$ that belong to the same scene as the image to be detected into the scene embedding $E_{scene}\;\in \;R^{256\times 64\times 64}$.
(3). $E_{image}$ and $E_{scene}$ are fused through the designed Feature Alignment and Fusion Module, and then fed into the Mask Decoder for mask prediction, yielding the segmentation results.

The SAID model is trained using the Industrial-5i dataset under a five-fold cross-validation setting, where each fold isolates one industrial scene for testing to evaluate cross-scene generalization. All input images and corresponding masks are resized to 1024 × 1024 and normalized to the [0, 1] range. To improve robustness, data augmentation techniques such as random horizontal flipping, brightness adjustment, and affine transformations are applied. During training, the Image Encoder is frozen to preserve pre-trained representations, while the Scene Encoder, Feature Alignment and Fusion Module, and Mask Decoder are optimized jointly in an end-to-end manner. The model is trained for 100 epochs using the Adam optimizer with a learning rate of $1\times 10^{-3}$ and the batch size is 16. A cosine annealing scheduler is employed to gradually reduce the learning rate. The total loss combines binary cross-entropy to handle the class imbalance in defect regions. All experiments are implemented in PyTorch 2.1 with mixed-precision training on an NVIDIA RTX 3090Ti GPU (Colorful, Shenzhen, China). During inference, the model performs segmentation using only the target image and a single-annotated product image pair from the same scene, without requiring any manual interaction or online fine-tuning.

### 3.2. Scene Encoder

**Figure 4:**
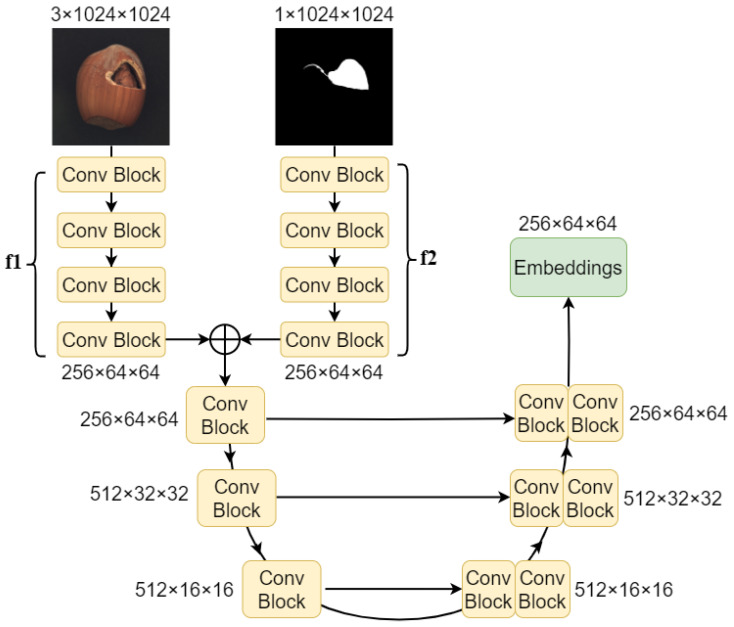
Scene Encoder. The single-annotated prompt–image pair I_sample and M_sample are encoded to achieve the scene embedding $E_{scene}$.

The Scene Encoder serves as a replacement for the complex human–model interaction mechanism in SAM, functioning as a prompt encoder. As illustrated in Figure 4, it is capable of encoding a pair of annotated scene prompts, namely a defective product image I_sample and its corresponding label mask image M_sample, into a scene embedding that carries domain expert knowledge. This embedding is then aligned and fused with the image embedding before being fed into the Mask Decoder. The Scene Encoder transforms the product input image $I\_sample\;\in \;R^{3\times H\times W}$ and its annotation $M\_sample\;\in \;R^{1\times H\times W}$ into a scene embedding $E_{scene}\;\in \;R^{bs\times 256\times 64\times 64}$. The input image to be detected $I_{input}\;\in \;R^{bs\times 3\times H\times W}$, after being processed by the Image Encoder, yields the image embedding $E_{image}\;\in \;R^{bs\times 256\times 64\times 64}$. Thanks to the robust feature extraction capabilities of the Image Encoder and the prompt encoding capabilities of the Scene Encoder, SAID exhibits excellent single-annotation cross-scene defect segmentation abilities.

Scene Encoder is a module designed for encoding scene information, based on CNNs (convolutional Neural Networks), and its architecture is inspired by the encoder–decoder structure of U-Net [41]. The U-Net architecture effectively extracts both local and global features from images, enabling the extraction of prior knowledge from a single-annotation prompt case. The scene prompt encoding component consists of two parts: the pre-encoder and the fusion encoder. The inputs are a product image $I\_sample\left(3\times 1024\times 1024\right)$ and its corresponding mask image $M\_sample\left(1\times 1024\times 1024\right)$. These two images are separately processed by two pre-encoders $f_{1}\left(\cdot \right)$ and $f_{2}\left(\cdot \right)$ to extract features, resulting in feature maps of size 256×64×64.

(1) $$\begin{array}{cc} F_{image} & =f_{1}\left(I\_sample\right),\\ F_{mask} & =f_{2}\left(M\_sample\right), \end{array}$$

$f_{1}\left(\cdot \right)$ represents the pre-encoder for the sample image I_sample, and $f_{2}\left(\cdot \right)$ represents the pre-encoder for the corresponding mask of the sample image M_sample. Both $f_{1}\left(\cdot \right)$ and $f_{2}\left(\cdot \right)$ have similar network architectures, consisting of four convolutional blocks. Each convolutional block $conv^{i}$ includes a 3×3 convolutional layer, a BN (Batch Normalization) layer, and a LeakyReLU activation function.

(2) $$conv^{i}=LeakyReLU\left(BN(conv\left(input)\right)\right),$$

*i* denotes the *i*-th convolutional block, with values ranging from 1 to 4. input refers to I_sample for $f_{1}\left(\cdot \right)$ or M_sample for $f_{2}\left(\cdot \right)$.

The feature maps $F_{image}$ and $F_{mask}$ are summed element-wise, and the resulting sum is processed by the fusion encoder f_fusion. The f_fusion consists of three convolutional blocks, each followed by a downsampling operation that reduces the feature map’s height and width to half of their original size while doubling the number of channels. Consequently, the shape of the input feature map transitions from $R^{256\times 64\times 64}$ to $R^{512\times 16\times 16}$.

(3) $$conv_{f\_fusion}^{i}=DownSamp\left(LeakyReLU\left(BN(conv\left(F_{image}+F_{mask})\right)\right)\right).$$

The scene prompt decoding part consists of three convolutional blocks, each preceded by an upsampling layer. The upsampling operation doubles the spatial dimensions of the feature map. Following each upsampling, a convolutional operation further extracts features while halving the number of channels. This transition results in the feature map’s shape changing from $R^{1024\times 8\times 8}$ to $R^{256\times 64\times 64}$.

(4) $$\begin{array}{cc} conv_{decoder}^{i}\left(x,y\right) & =LeakyReLU\left(conv\left(concat\left(UpSamp(x),y\right)\right)\right), \end{array}$$

**Figure 5:**
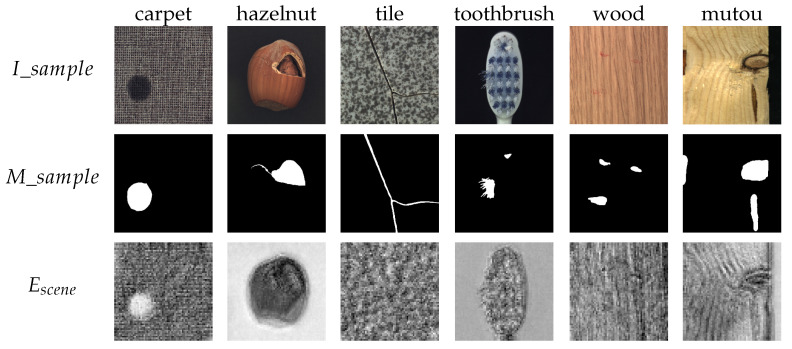
Visualization of single-annotated prompt–image pairs I_sample, M_sample, and scene embedding $E_{scene}$.

UpSamp(x) is implemented using nearest-neighbor interpolation, which enlarges the spatial dimensions of the low-resolution feature map through interpolation. This is followed by a 1×1 convolutional layer to adjust the dimensions. In each convolutional operation of the decoder, after the upsampling and dimensionality reduction, the output is concatenated with the corresponding convolutional block output from the encoder. This concatenation aids the model in better feature extraction. Ultimately, after the encoding–decoding process, a scene embedding $E_{scene}\;\in \;R^{bs\times 256\times 64\times 64}$ is obtained, which has the same shape as the image embedding $E_{image}$. The final results are shown in Figure 5.

### 3.3. Feature Alignment and Fusion Module

The $E_{image}$ output by the Image Encoder naturally exhibits misalignment in the feature space with the $E_{scene}$ output by the Scene Encoder. A direct approach to address this issue would be to fine-tune the Image Encoder. However, due to the limited amount of data for downstream tasks, retraining could easily lead to overfitting and fail to fully leverage the zero-shot emergence capabilities of SAM. To address this, we introduce a Lightweight Feature Alignment and Fusion Module, which includes Neck modules and a Lightweight Fusion module, as shown in Figure 3. Before being fused with $E_{scene}$, $E_{image}$ undergoes alignment through the Neck modules, followed by feature fusion through the Lightweight Fusion module.

(5) $$\begin{array}{cc} E_{image}^{\prime } & =\mathrm{ReLU}\left(W_{image\_2}\cdot \mathrm{ReLU}\left(W_{image\_1}\cdot E_{image}+b_{image\_1}\right)+b_{image\_2}\right),\\ E_{scene}^{\prime } & =\mathrm{ReLU}\left(W_{scene\_2}\cdot \mathrm{ReLU}\left(W_{scene\_1}\cdot E_{scene}+b_{scene\_1}\right)+b_{scene\_2}\right), \end{array}$$

$W_{image\_1}$, $b_{image\_1}$, $W_{image\_2}$, and $b_{image\_2}$ represent the weight matrices and bias vectors for the first and second layers of the MLP processing $E_{image}$, respectively. Similarly, $W_{scene\_1}$, $b_{scene\_1}$, $W_{scene\_2}$, and $b_{scene\_2}$ denote the weight matrices and bias vectors for the first and second layers of the MLP processing $E_{scene}$. The symbol ReLU(·) represents the nonlinear activation function ReLU.

**Figure 6:**
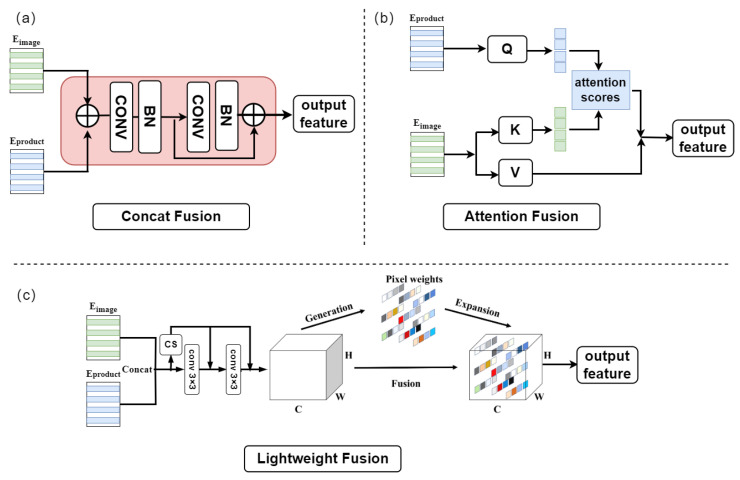
The structure of different fusion modules. (**a**) Concat fusion mechanism. (**b**) Cross-attention fusion mechanism. (**c**) Lightweight 3D Fusion Mechanism.

To align and fuse the $E_{image}^{\prime }$ output by the Image Encoder with the $E_{scene}^{\prime }$ output by the Scene Encoder in the feature space, multiple experiments were conducted. The Neck module designed in this paper is a single-layer MLP that follows both the Image Encoder and the Scene Encoder. This module does not alter the shape of the embedding but ensures that the image embedding and scene embedding are aligned in the feature dimension, thereby reducing the likelihood of suboptimal segmentation results due to feature misalignment introduced by the Scene Encoder module. After introducing the Neck modules to align the image embedding and scene embedding, the Lightweight Fusion module further integrates these embedding. Inspired by [42], the Lightweight Fusion module designed in this paper is a Lightweight feature fusion module that incorporates a pixel-level 3D attention mechanism. As shown in Figure 6c, the $E_{image}^{\prime }$ and $E_{scene}^{\prime }$ from Equation (5) are concatenated and then processed through two simple convolutional operations followed by a pixel-level attention module for feature fusion. The two convolutional layers are connected with skip connections that concat with the input, and finally, a parameter-free attention mechanism is used to further fuse the bound features.

(6) $$x=conv(concat\left(E_{image}^{\prime },E_{scene}^{\prime }\right)),$$

conv(·) represents a two-layer convolutional operation. The bound features from Equation (6) are further fused through Equation (7).

(7) $$E_{feature}=x\times \sigma \left(\frac{{\left(x-\mu \right)}^{2}}{4\left(\frac{\sum_{i=1}^{n}{\left(x_{i}-\mu \right)}^{2}}{n}+\epsilon \right)}+0.5\right),$$

μ denotes the mean of the vector *x*, which is computed using the formula $\mu =\frac{1}{n}\sum_{i=1}^{n}x_{i}.$ Here, $x_{i}$ represents the individual feature point within the vector *x*. *n* represents the number of effective pixel values within the vector *x*. This is determined by the expression n=w×h−1, where *w* and *h* are the width and height of the image, respectively. The subtraction of 1 accounts for any potential boundary effects or excluded pixels. ϵ is a small constant introduced to prevent division by zero, ensuring numerical stability in the calculations. In the conducted experiments, this constant is set to $1\times 10^{-4}$. The symbol σ(·) signifies the Sigmoid activation function, which is defined as $\sigma \left(z\right)=\frac{1}{1+e^{-z}}.$ σ(·) maps the real-valued number to a value between 0 and 1, making it particularly useful for binary classification tasks and as an activation function in neural networks. Finally, as shown in Equation (8), the fused feature $E_{feature}$ is decoded by the Mask Decoder to produce the output predicted mask image $M_{Output}$.

(8) $$M_{Output}\;=\;dec_{Mask}\left(E_{feature}\right),$$

where $dec_{Mask}\left(\cdot \right)$ is the Mask Decoder of SAID.

### 3.4. Loss Function

For the network SAID proposed, the loss function is the cross-entropy loss between the predicted mask and the GT (Ground Truth) mask, which is a commonly used loss function in segmentation networks, denoted as $L_{seg}$. The cross-entropy loss $L_{seg}$ quantifies the difference between the predicted mask and the true label mask.

(9) $$L_{seg}=-\frac{1}{N}\sum_{i=1}^{N}\left[y_{i}\log\left(p_{i}\right)+\left(1-y_{i}\right)\log\left(1-p_{i}\right)\right],$$

$L_{seg}$ evaluates the model’s performance by calculating the difference between the model’s predicted probability distribution and the true probability distribution at each pixel. Here, N=H×W represents the total number of pixels, $y_{i}$ is the true label of the *i*-th pixel, and $p_{i}$ is the model’s predicted value for the *i*-th pixel.

## 4. Experiments

### 4.1. Setup

**Datasets** To validate the generality of our approach, we use the Industrial-5i dataset compiled in [43]. This dataset includes images from 20 different industrial scenes sourced from MVtec-AD [44], KolektorSDD [45], Magnetic Tile Defect [46], RSDDs [47], and BSData [48]. The Industrial-5i dataset was divided into four folds, and the network was trained and validated using a cross-validation approach. Specifically, one fold of the scenes was selected as the test set to evaluate the model’s generalization capability, while the remaining scene categories served as the training set to train the network model. During training, the training set data underwent data augmentation to expand to five times its original size. Detailed information about the dataset can be found in Table 1.

**Evaluation Metrics** Following prior research [43,49], we adopted mIoU as the primary evaluation metric for our experiments. mIoU is a widely recognized metric in the field of image segmentation, used to quantify the overlap between the predicted segmentation and the ground truth segmentation. For a single category, the intersection-over-union (IoU) is defined as the ratio of the area of intersection between the predicted segmentation and the ground truth mask to the area of their union. mIoU is the average of IoU across all categories. The formula for calculating mIoU is as follows,

(10) $$\mathrm{mIoU}=\frac{1}{2}\sum_{c=0}^{1}\frac{TP_{c}}{FP_{c}+TP_{c}+FN_{c}}.$$

Here, mIoU represents the mean intersection-over-union for the two classes (0 and 1). $TP_{c}$, $FP_{c}$, and $FN_{c}$ denote the True Positives, False Positives, and False Negatives for class *c*, respectively. The IoU for each class is computed as the ratio of the intersection area to the union area between the predicted and ground truth labels. The mIoU is the average of these two IoU values. The mIoU ranges from 0 to 1, with higher values indicating superior segmentation performance in binary semantic segmentation tasks.

**Implementation details** We use the loss function (10) to supervise the training of our network SAID. To address the issue of the large number of parameters in Image Encoder, we introduced a Lightweight EfficientSAM [50] Image Encoder (based on different versions of ViT) for comparative experiments. During training, the parameters of the Image Encoder remained frozen and were not updated. All experiments used the representative Adam optimizer with an initial learning rate set to $1\times 10^{-3}$ and the batch size set to 16. We employed a warm-up learning rate strategy combined with cosine decay [51] for adjustment. The experiments were conducted on an NVIDIA RTX 3090Ti GPU using the PyTorch 2.1 framework for training.

### 4.2. Main Results

#### 4.2.1. Cross-Scene One-Shot Segmentation

**Figure 7:**
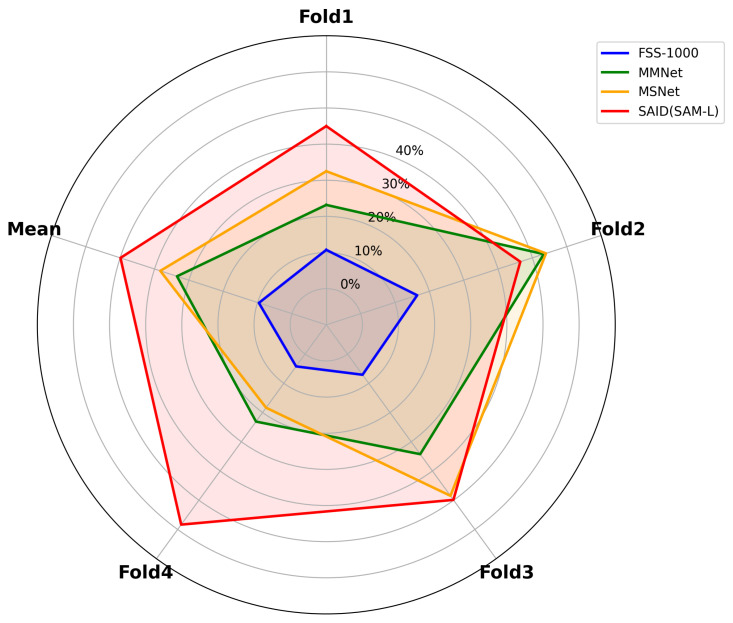
Visualization of mIoU performance for single segmentation on the Industrial-5i dataset.

For the cross-scene one-shot segmentation task, we conduct four sets of cross-validation experiments on the Industrial-5i dataset mentioned in Section 4.1. The Industrial-5i dataset consists of four folds, and we sequentially select one fold as the validation set, with the remaining three folds serving as the training set. The training set underwent data augmentation, expanding its size to five times the original. To investigate the impact of different Backbone models on the segmentation performance of SAID, we study the Image Encoder of SAM in two versions: ViT-L and ViT-B. Additionally, following the work of EfficientSAM [50] on distilling a Lightweight version of SAM, we also examine the Image Encoder of EfficientSAM in two versions: ViT-T and ViT-S. Table 2 presents the experimental results on the Industrial-5i dataset, with SAID compared to advanced single-annotation prompt segmentation models. Among them, FSS-1000 [52] serves as an important benchmark dataset in the few-shot segmentation field, providing a unified platform for evaluating the generalization performance of subsequent models. Based on this task, MMNet$\tilde{c}$itewu2021learning enhances the semantic correlation between support and query images by introducing a multi-level memory mechanism, improving the model’s contextual understanding ability. MSNet [25] emphasizes the fusion and interaction of multi-scale features to adapt to the diversity of target sizes and shapes. SegGPT [26] transforms image segmentation tasks into sequence-to-sequence generation problems, achieving strong generalization ability under zero and few sample conditions through pixel-level modeling of image content. The results show that SAID performs exceptionally well in industrial defect detection, achieving higher mIoU metrics compared to other one-shot segmentation methods. Under the one-shot setting, our method improved the mIoU by 0.87 and 5.79 compared to MSNet [25] and SegGPT [26], respectively. The performance comparison between them can be clearly seen in Figure 7.

To evaluate the reliability of the models, we measured their inference time on the RTX 3090 Ti, as summarized in Table 3. It can be observed that SAID requires a relatively longer time for full inference. This is primarily due to its Image Encoder, which is based on a computationally intensive Vision Transformer (ViT). However, if the input image is pre-encoded into an image embedding, the inference time per image can be significantly reduced to only 15–20 ms, which is comparable to that of other methods.

**Figure 8:**
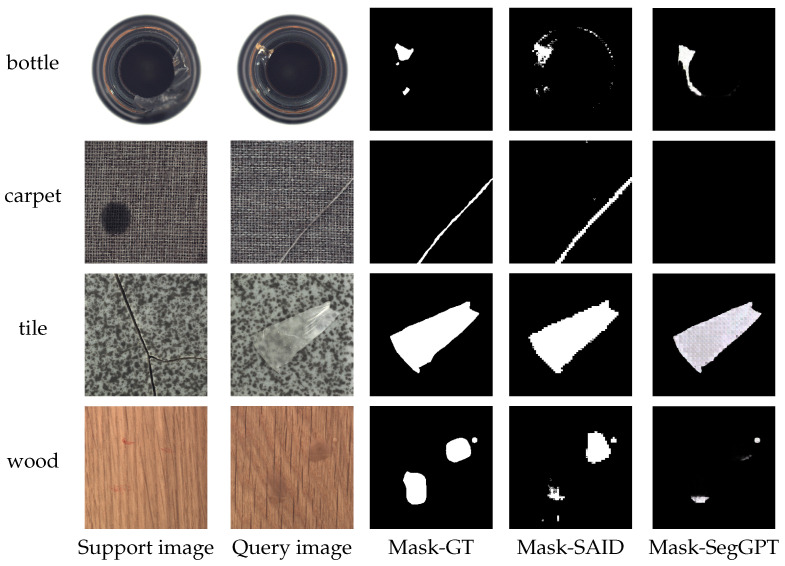
Visual comparison of one-shot segmentation results of SAID and SegGPT on various industrial scenes.

Traditional one-shot or few-shot segmentation methods typically rely on comparison with a support image to locate the target (appearance defects) in the query image. The core task of these methods is to learn features from one or a few normal scene images for defect detection in the query image. In contrast, SAID outperforms other one-shot segmentation models because it leverages scene-consistent prompts derived from the same industrial environment, enabling better alignment between support and query images. This reduces domain gaps and improves defect localization accuracy. Figure 8 demonstrates the defect segmentation results of our method on some industrial images. Even in situations with complex and varied image textures and colors, our method can generate accurate defect segmentation maps, showing its effectiveness and robustness in practical applications.

#### 4.2.2. Supervised Experiment

**Figure 9:**
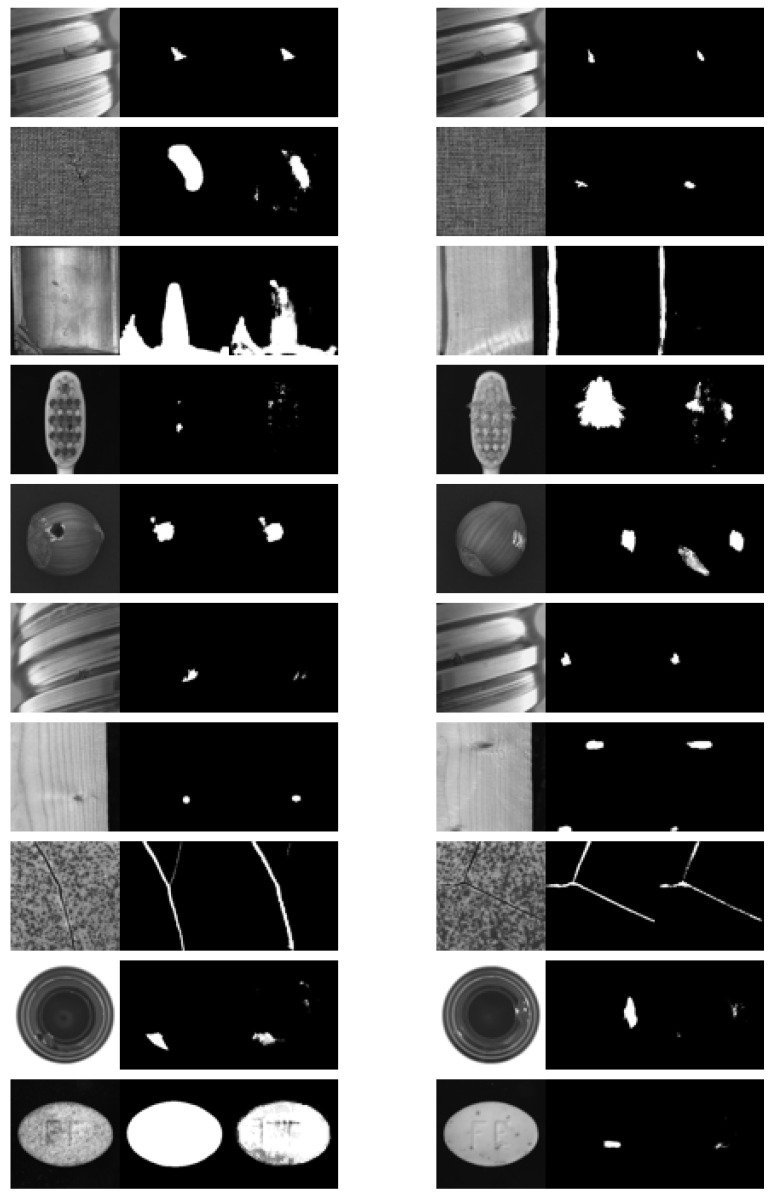
Performance results of SAID on various industrial datasets.

The goal of one-shot learning is to train a model that can quickly adapt to new categories. However, due to the limited amount of training data in one-shot learning, the model may be at risk of overfitting. To further validate the capabilities of SAID, we conduct a supervised experiment to verify the model’s performance on specific data (single scene category data). We perform supervised training and testing on 15 industrial scenes from MVtec-AD [44], with 80% of the images from each industrial scene category used for training and the remaining 20% for testing. We compare our model with three prompting models of SAM, and the results are shown in Table 4. Compared with SAM, SAID increases the mIoU metric to 0.725, while SAM’s mIoU strong human prior knowledge box prompting is 0.635. The final results are shown in Figure 9.

In Table 4, the everything mode used by SAM involves generating a uniform grid point prompt (m×m) on the image to be detected, where the value of *m* is set to 32 by default; the point mode selects a positive prompt point (a prompt point in the defect area); and the box mode uses the outer rectangle box of the GT as the prompt box, which is a very precise human prior. It can be clearly seen from Table 4 that, except for a few scenes (such as capsule and transistor), our model’s segmentation capabilities in specific scenes can basically reach or even surpass the segmentation capabilities of the SAM with strong human prior knowledge in box mode.

To further evaluate the practical usability of our proposed method, we compare the interaction efforts required by SAM and SAID in generating segmentation masks. For SAM, producing an accurate segmentation result generally demands manual input for each image. In point-based prompting, users typically need to provide 3–5 point clicks, and the interaction process takes approximately 8–12 s per image, depending on the complexity of the defect and the precision of the user input. In box-based prompting, segmentation can often be achieved with 1–3 bounding boxes, requiring around 3–5 s per image. However, both modes still involve manual interaction and iterative refinement to achieve acceptable segmentation quality.

### 4.3. Ablation Experiment

We conduct ablation experiments based on Table 5, following the conclusions drawn from Section 4.2. To explore the impact of the Scene Encoder module and the Feature Alignment and Fusion Module on the performance of the SAID model, we construct ablation experiments. The experimental data used the four sets of data from Table 1, and the evaluation metric was the mIoU of the model’s one-shot segmentation results. Among them, $SAM_{Everything}$ is the everything mode of SAM, the same as in Section 4.2.2. Using this segmentation method, the model outputs a large number of masks, and we select the mask with the highest overlap with the GT as the final output for comparison. FT (no prompt) removes the prompter encoder from SAM and fine-tunes the Mask Decoder using the industrial image data from the remaining three folds to adapt it to the distribution of industrial images. At the same time, it specifies the output of a single mask, avoiding multiple outputs and the complex post-processing operations. The Scene Encoder proposed in this paper provides scene information to assist in segmentation through a pair of example images. Finally, on this basis, the Feature Alignment and Fusion Module (FA-F) is added.

From Table 5, it can be seen that although the “$SAM_{Everything}$ + Post-processing” mode achieved high mIoU in some cases (such as on fold1 data), the images segmented by $SAM_{Everything}$ have multiple mask outputs, and it is necessary to calculate the best mask as the result by comparing multiple outputs with the GT. However, in real detection needs, there is no GT detection process, and this method is not applicable. In contrast, our model benefits from the Scene Encoder and Lightweight Fusion, and the detection effect has exceeded the $SAM_{Everything}$ mode, while also achieving automatic detection without the need for subsequent processing operations, making it a more ideal detection method.

The efficient fusion of $E_{image}$ and $E_{scene}$ is crucial for SAID to enhance the model’s segmentation performance. This paper compares the impact of different fusion mechanisms on model performance, primarily contrasting: (a) Concat Fusion; (b) Attention Fusion; and (c) Lightweight Fusion. These three fusion mechanisms are illustrated in Figure 6. Concat Fusion involves concatenating $E_{image}$ and $E_{scene}$ and then passing them through a convolutional module for fusion output. Attention Fusion employs a cross-attention mechanism, using $E_{scene}$ as the query Q and $E_{image}$ as the key *K* and the value *V*, to fuse features by extracting useful information through cross-attention. Lightweight Fusion concatenates the features through a simple convolution and then fuses them through the 3D attention mechanism in Equation (7).

The results in Table 6 clearly indicate that the superiority of the proposed Lightweight 3D Fusion Mechanism can be attributed to its design, which explicitly aligns the scene prompt features with the query image features in both spatial and semantic dimensions using a low-cost bottleneck structure. Unlike simple concatenation or element-wise addition, the 3D fusion incorporates both channel-wise and token-wise interactions, enhancing the feature consistency between prompt and query inputs. Therefore, this paper selects this fusion mechanism to fuse $E_{image}$ and $E_{scene}$ embedding, aiming to achieve efficient feature fusion and improve the model performance.

## 5. Conclusions

This study addresses the limited generalization of traditional deep learning-based defect detection models and the practical shortcomings of the general-purpose SAM model in industrial downstream tasks, including its reliance on manual interaction, lack of automatic segmentation capability, and complex post-processing. To overcome these issues, we propose SAID—a single-annotation scene prompt segmentation framework. SAID integrates a Scene Encoder to extract contextual scene-level embeddings from annotated product image pairs and introduces a Lightweight Fusion module to effectively combine image and scene embeddings. By leveraging scene-specific prior knowledge and fine-tuning on multi-domain defect data, SAID achieves automatic and accurate segmentation across diverse industrial environments.

While SAID demonstrates strong segmentation performance, certain limitations remain. First, the model relies on a representative annotated prompt image from the same scene, which may not always be readily available in real-world online inspection scenarios involving unseen or evolving product types. Second, although the model eliminates the need for real-time human interaction, its inference speed is limited by the high computational cost of the frozen Image Encoder inherited from SAM, making it less suitable for latency-sensitive or edge device deployment. Additionally, SAID may experience performance degradation under severe occlusion, extreme lighting conditions, or highly ambiguous defect textures.

Future research will address these challenges by exploring a dynamic prompt selection mechanism capable of automatically identifying the most relevant support samples from a historical defect database, reducing reliance on manually chosen prompt images. We also aim to incorporate online adaptive learning to continuously refine the model as new product types or defect styles emerge. Furthermore, we plan to compress and distill the model to enable real-time deployment on edge devices in resource-constrained environments. A more comprehensive robustness evaluation will also be conducted to systematically assess performance under varied industrial conditions, including low-contrast defects, noisy backgrounds, and camera misalignments.

## Figures and Tables

**Table 1:** Classes and the original corresponding number of images in the industry-5i dataset.

Fold1	Wood	Pill	BSD	Railway	Toothbrush
	60	141	426	94	30
Fold2	Leather	Mutou	Metal-Nut	Kolektor-SSD2	Bottle
	92	1838	70	436	63
Fold3	Carpet	Hazelnut	Phone	Tile	Grid
	89	70	100	84	57
Fold4	Magnetic Tile	Capsule	Cable	Kolektor-SSD	Zipper
	392	109	92	522	119

**Table 2:** The mIoU performance of one-shot segmentation on Industrial-5i dataset.

Methods	Fold1	Fold2	Fold3	Fold4	Mean
FSS-1000 [52]	10.37	13.23	8.54	7.11	9.81
MMNet [23]	16.59	31.66	22.12	16.55	21.73
MSNet [25]	21.25	**31.98**	29.24	14.18	24.16
SegGPT [26]	**31.16**	22.98	28.69	33.47	29.08
SAID(EfficentSAM-T)	24.67	27.69	27.66	20.41	25.61
SAID(EfficentSAM-S)	26.01	27.42	28.72	24.37	26.13
SAID(SAM-B)	25.79	26.68	29.64	26.09	26.80
SAID(SAM-L)	27.49	28.24	**29.94**	**34.17**	**29.96**

**Table 3:** Model comparison on RTX 3090 Ti.

Model	Inference Time (ms)	Params (M)	Architecture Characteristics
FSS-1000	30–60	45	ResNet-101 backbone with prototype matching
MMNet	10–20	2.1	Lightweight multi-scale CNN with attention
MSNet	8–15	10	Multi-scale autoencoder with memory bank
SAID (SAM-B)	250–350	91	Vision Transformer-Base with mask decoder
SAID (SAM-L)	500–800	308	Vision Transformer-Large with mask decoder
SAID (pre-encoded)	15–20	308	Vision Transformer-Huge with mask decoder

**Table 4:** SAID performance in supervised training on the MVtec dataset, with mIoU as the evaluation metric. “Human” indicates whether manual interaction is required.

Category	${\mathrm{SAM}}_{\mathrm{Everything}}$		${\mathrm{SAM}}_{\mathrm{Point}}$		${\mathrm{SAM}}_{\mathrm{Box}}$		SAID(ours)	
	**mIoU**	**Human**	**mIoU**	**Human**	**mIoU**	**Human**	**mIoU**	**Human**
bottle	0.298	N	0.489	Y	0.675	Y	**0.831**	N
cable	0.410	N	0.560	Y	**0.676**	Y	0.589	N
capsule	0.316	N	0.444	Y	**0.562**	Y	0.482	N
carpet	0.045	N	0.296	Y	0.475	Y	**0.673**	N
grid	0.144	N	0.265	Y	**0.526**	Y	0.494	N
hazelnut	0.439	N	0.589	Y	0.705	Y	**0.891**	N
leather	0.291	N	0.485	Y	0.631	Y	**0.762**	N
metal_nut	0.355	N	0.696	Y	0.671	Y	**0.894**	N
pill	0.374	N	0.570	Y	**0.743**	Y	0.703	N
screw	0.208	N	0.455	Y	0.635	Y	**0.797**	N
tile	0.337	N	0.730	Y	0.726	Y	**0.835**	N
toothbrush	0.263	N	0.446	Y	0.735	Y	**0.877**	N
transistor	0.324	N	0.325	Y	**0.445**	Y	0.339	N
wood	0.176	N	0.325	Y	0.650	Y	**0.834**	N
zipper	0.149	N	0.257	Y	0.588	Y	**0.806**	N
Mean	0.279	N	0.468	Y	0.635	Y	**0.725**	N

**Table 5:** Ablation experiment results, FT stands for fine-tuned Mask Decoder, and FA-F refers to the Light Weight Fusion module proposed.

	FT	Scene Encoder	FA-F	Fold1	Fold2	Fold3	Fold4	Mean
$SAM_{Everything}$	✗	✗	✗	**33.70**	24.86	24.36	20.01	25.73
FT (No prompt)	✓	✗	✗	18.65	20.42	26.43	18.42	20.98
Scene Encoder	✓	✓	✗	23.74	24.12	29.58	25.16	25.65
Ours	✓	✓	✓	27.49	**28.24**	**29.94**	**34.17**	**29.29**

**Table 6:** The impact of various fusion modules on model performance, with the evaluation metric being mIoU.

Fusion Modules	Fold1	Fold2	Fold3	Fold4	Mean
Concat Fusion	22.65	24.43	**30.54**	23.13	25.19
Attention Fusion	25.62	25.61	29.11	26.69	26.76
Lightweight Fusion	**27.49**	**28.24**	29.94	**34.17**	**29.95**

## Data Availability

Data sharing is not applicable to this article as no new data were created or analyzed in this study.
